# Supplementary figures and images for: MLL1 is required for maintenance of intestinal stem cells
Source: PLoS Genet. 2021 Dec 3;17(12):e1009250. doi: 10.1371/journal.pgen.1009250 (PMC8641872; doi:10.1371/journal.pgen.1009250)

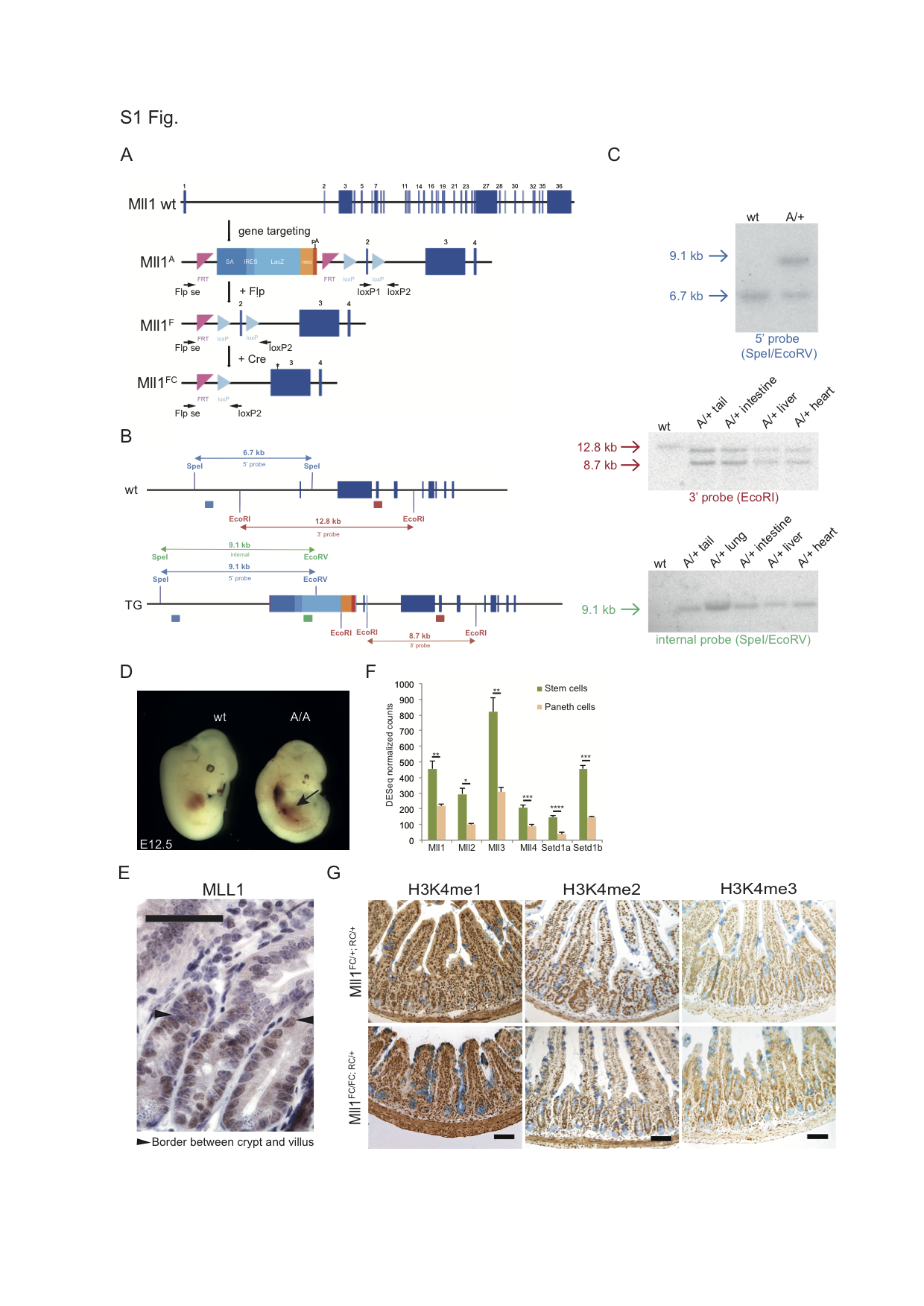

Supplement: S1 Fig — (A) Diagram of the Mll1 gene with numbered exons and the multipurpose allele (Mll1A). This allele is converted to Mll1F upon FLP recombination. Cre recombination leads to excision of the frameshifting exon 2 generating the conditional mutant allele (Mll1FC). Genotyping primers are depicted for the downstream loxP site (loxP1 –loxP2) and for Flp recombination (Flp se–loxP2). SA = splice acceptor, IRES = internal ribosome entry site, pA = polyadenylation signal, lacZ-neo = β-galactosidase and neomycin resistance gene, * depicts premature stop codon. (B) Schematic representation of the Southern blot strategy. For identifying correct targeted events in the Mll1 locus, Southern blot analysis employed 5’ (blue box), 3’ (red box) and internal LacZ (green box) probes. (C) Southern blot analysis with 5’ external probe (wt and correctly targeted ESC clone). Southern blot analysis with 3’ external and LacZ internal probes (wt ESCs and organs from an adult Mll1A/+ mouse). (D) Dissected embryos from Mll1A/+ intercrosses at E12.5. Mll1A/A embryos had a pale liver (marked by arrow). (E) Antibody staining (brown) shows that MLL1 is expressed in crypts and TA compartment of the small intestine but is absent in the villus (hematoxylin, purple). Scale bar 50 μm. (F) Normalized RNA-sequence counts for Mll1/Kmt2a, Mll2/Kmt2b, Mll3/Kmt2c, Mll4/Kmt2d, Setd1a/Kmt2f and Setd1b/Kmt2g in ISCs (eGFPhigh) and Paneth cells sorted from Lgr5-eGFP-CreERT2 mice. Mean+s.d. is shown; n = 4; *p<0.05, **p<0.01, ***p<0.001, ****p<0.0001, Student’s t test. (G) Antibody stainings of H3K4me1, H3K4me2 and H3K4me3 are comparable in Mll1FC/+; RC/+ and Mll1FC/FC; RC/+ intestinal sections. Scale bars are 100 μm. (TIF) [file pgen.1009250.s001.tif]

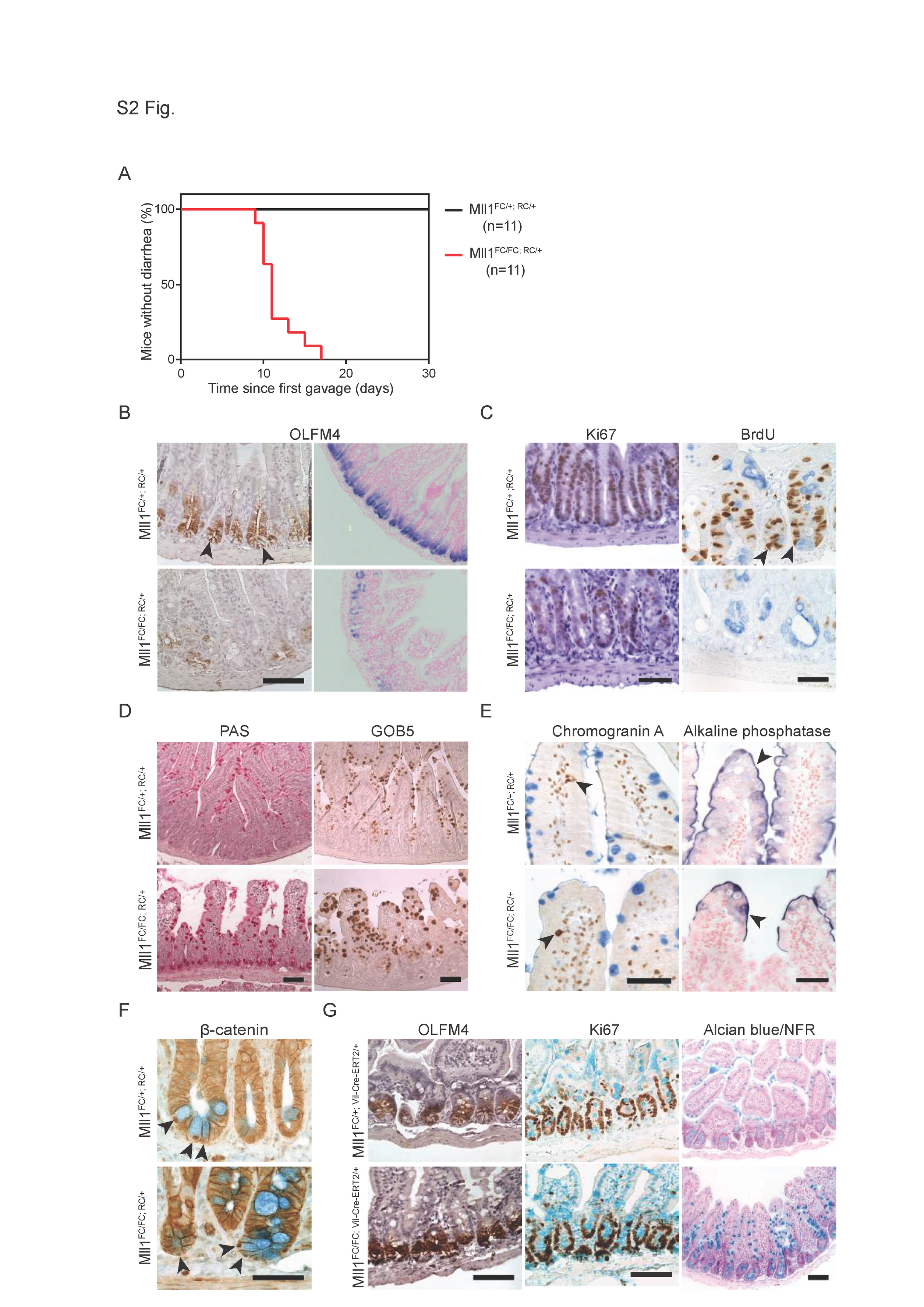

Supplement: S2 Fig — (A) Kaplan-Meier analysis for the onset of diarrhea. Tamoxifen was given by gavage for 6 days to Mll1F/+; RC/+ (n = 11) and Mll1F/F; RC/+ (n = 11) mice. The first day of tamoxifen gavage was day zero. While all mice with the genotype Mll1FC/+; RC/+ remained healthy, all Mll1FC/FC; RC/+ mice developed diarrhea with a median of 11 days. (B) Antibody stain (left panels) and in situ hybridization (right panels) to visualize OLFM4/Olfm4 in intestinal sections. Arrowheads point towards ISCs. Scale bar 100 μm. (C) Proliferative activity visualized by both Ki67 stain and BrdU incorporation in intestinal sections. Arrowheads point towards proliferative ISCs. Scale bars are 50 μm. (D) PAS staining and GOB5 antibody stain to visualize goblet cells in intestinal sections. Scale bars are 100 μm. (E) Chromogranin A and alkaline phosphatase staining to visualize enteroendocrine cells and enterocytes respectively. Arrows point to enteroendocrine cells (brown cytoplasmic stain) in the villi. Blue enterocytes covering the villi are marked by arrowheads. Scale bars are 100 μm for chromogranin A and 50 μm for alkaline phosphatase. (F) Nuclear β-catenin is comparable between the two different genotypes. Arrowheads point at β-catenin positive nuclei. Scale bar is 50 μm. (G) Left and middle panels; antibody stainings to visualize OLFM4 and Ki67 expression in Mll1FC/FC; Vil-Cre-ERT2/+ intestine compared to controls, four weeks after the first gavage. Hematoxylin was used as a counterstain for OLFM4 and alcian blue was used for Ki67 IHC. Right panel; alcian blue staining of goblet cells with NFR to stain nuclei, four weeks after the first gavage. Scale bars are 100 μm. (TIF) [file pgen.1009250.s002.tif]

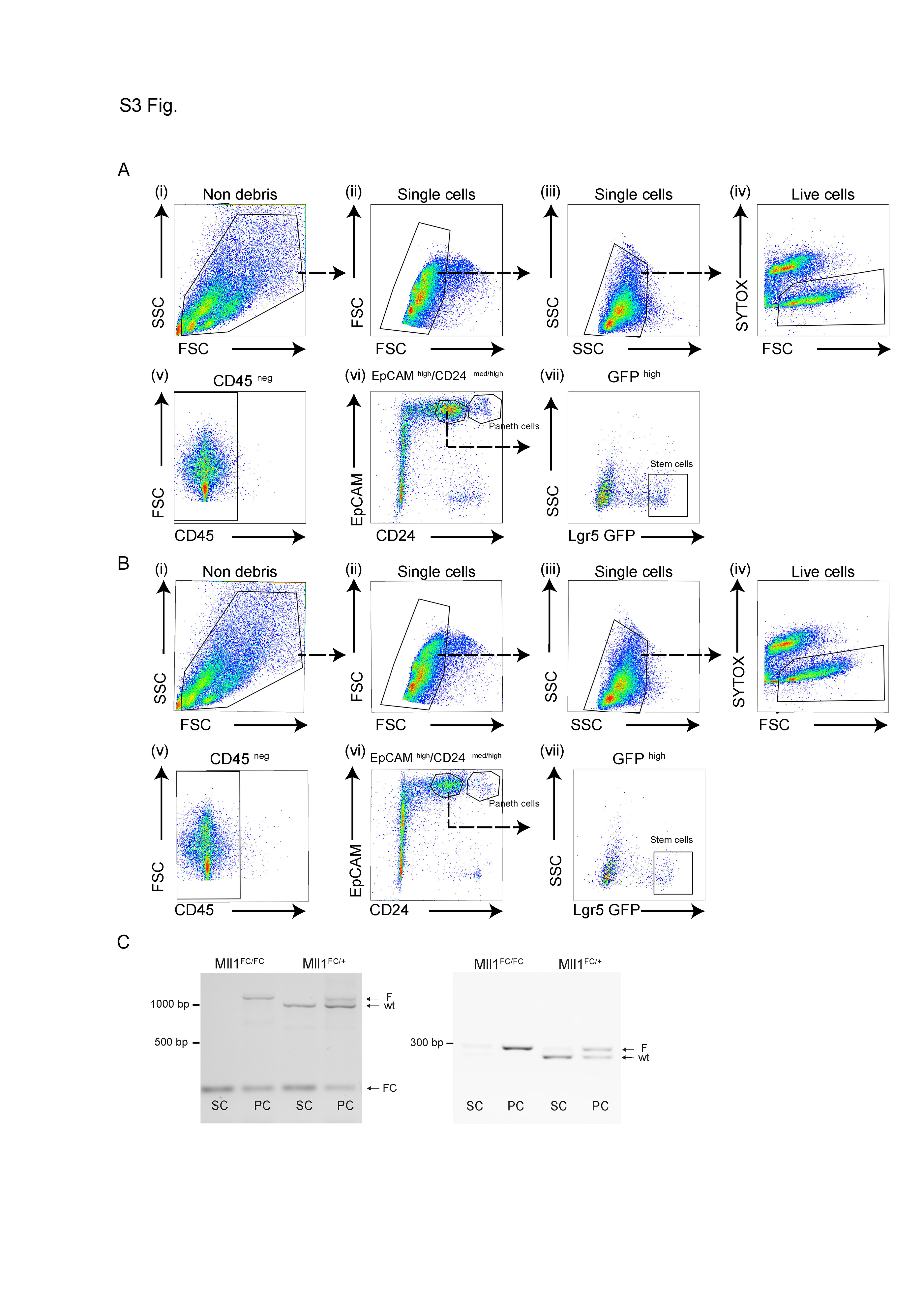

Supplement: S3 Fig — Flow sorting on (A) Mll1FC/+; Lgr5-eGFP-CreERT2/+ and (B) Mll1FC/FC; Lgr5-eGFP-CreERT2/+ single cell suspension of crypts. Briefly, the consecutive gating steps were applied: (i)–(iii) Definition of the population of interest by exclusion of debris based on size (FSC), granularity (SSC) and the selection for single cells; (iv) Exclusion of dead cells that incorporated the nucleic acid stain SYTOX blue; (v) Depletion of CD45pos population; (vi) Definition of Paneth (EpCAMhigh/CD24high) cell population by plotting EpCAM vs CD24 fluorescence; (vii) EpCAMhigh/CD24med cell population was gated to discriminate the stem cell population (GFPhigh). (C) Stem cells (SC) and Paneth cells (PC) from Mll1FC/+; Lgr5-eGFP-CreERT2/+ and Mll1FC/FC; Lgr5-eGFP-CreERT2/+ mice 4 days after tamoxifen induction were checked for recombination. Left panel; PCR genotyping was using primers (as shown in S1A Fig) upstream of the 5’ FRT site and downstream of the 3’ loxP site identified the Mll1F band at 1084 bp, the wild type band a 933 bp and the Mll1FC band at 186 bp. Right panel; primers flanking the 3’ loxP site identified the Mll1F band at 297 bp and the wild type band at 251 bp. (TIF) [file pgen.1009250.s003.tif]

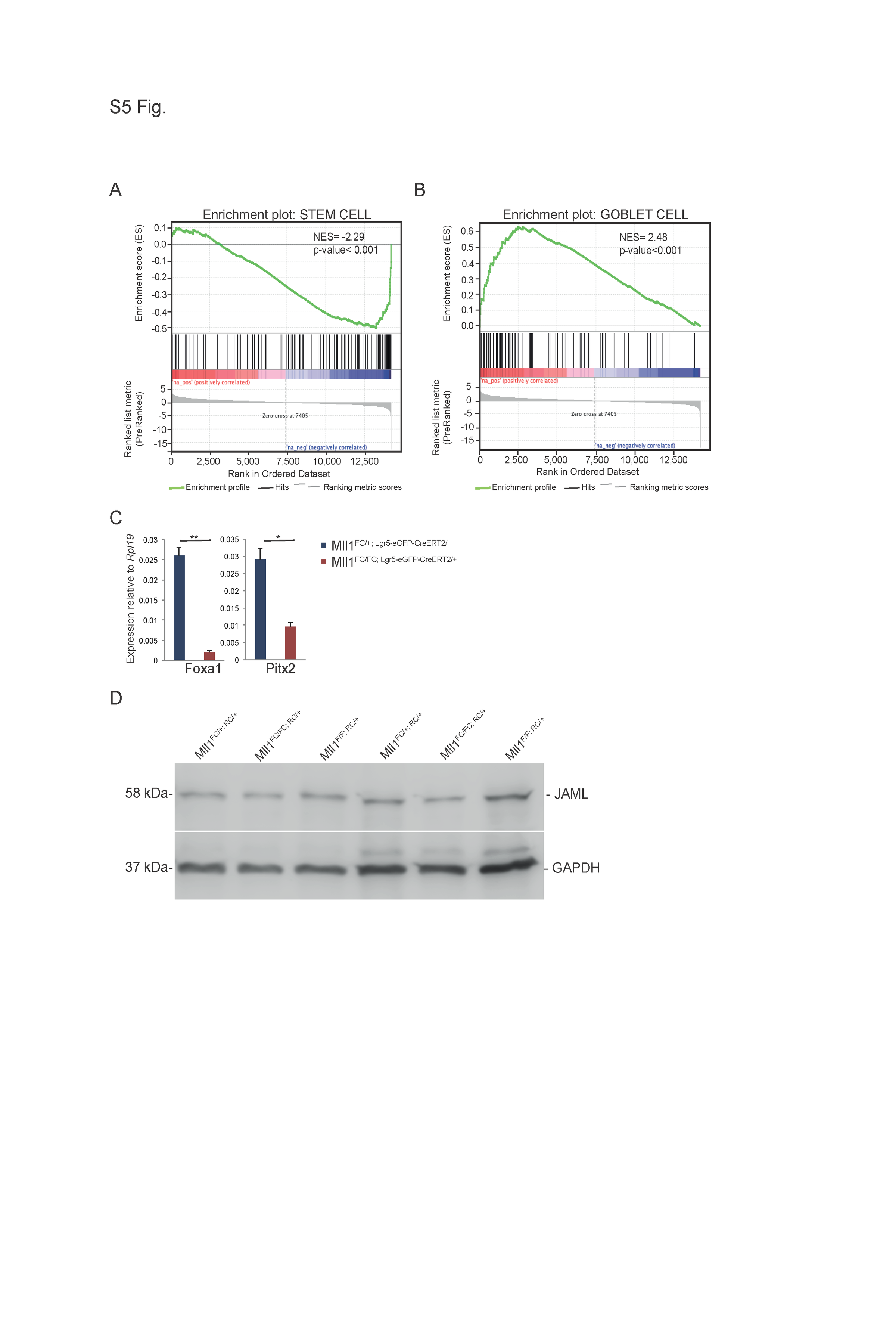

Supplement: S5 Fig — (A) (B) GSEA shows significant negative or positive correlation of genes from the stem (A) and goblet cell (B) signature gene set in Mll1FC/FC; Lgr5-eGFP-CreERT2/+ ISCs compared to control ISCs 10 days after tamoxifen induction was completed. The signature gene sets originate from [87]. NES: normalized enrichment score. (C) To validate RNA-seq results qRT-PCR was performed for selected genes on cDNA from Mll1FC/+; Lgr5-eGFP-CreERT2/+ and Mll1FC/FC; Lgr5-eGFP-CreERT2/+ sorted stem cells 4 days after tamoxifen induction was completed. Mean+s.d. is shown; n = 3; *p<0.05, **p<0.01, Student’s t test. (D) Western blot analysis shows no change in JAML protein levels in Mll1FC/+; RC/+, Mll1FC/FC; RC/+ and Mll1F/F; RC/+ organoids (n = 2). GAPDH is the loading control. (TIF) [file pgen.1009250.s005.tif]

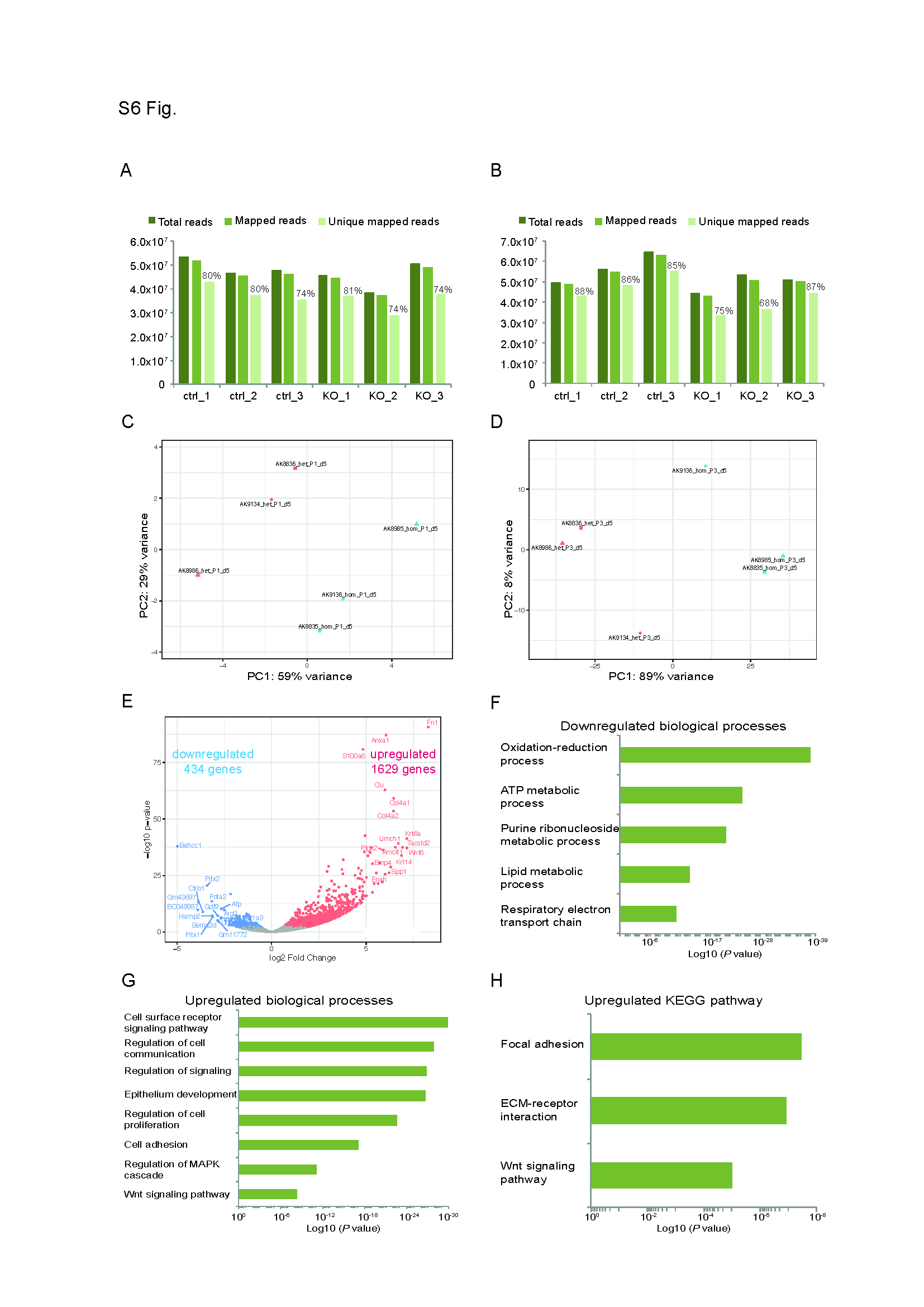

Supplement: S6 Fig — (A, B) Intestinal organoids were analyzed from Mll1FC/+; RC/+ (ctrl) (n = 3) and Mll1FC/FC; RC/+ (KO) (n = 3) mice. Mapability of reads after passage 1 (A) and passage 3 (B). (C, D) Principal-component analysis (PCA) was performed on intestinal organoids after passage 1 (C) and passage 3 (D). PCA is based on mRNA changes for the top 500 most diverse genes. (E) mRNA profiling of Mll1FC/+; RC/+ and Mll1FC/FC; RC/+ intestinal organoids after passage 3. Volcano plot visualizing the log2-fold change differences according to expression levels. Blue and red dots represent significant down- and upregulated DEGs, respectively, at a 5% FDR. (F-H) Enriched terms of biological processes and pathways downregulated (F) and upregulated (G, H) using DAVID GO/BP/FAT and KEGG database. (TIF) [file pgen.1009250.s006.tif]

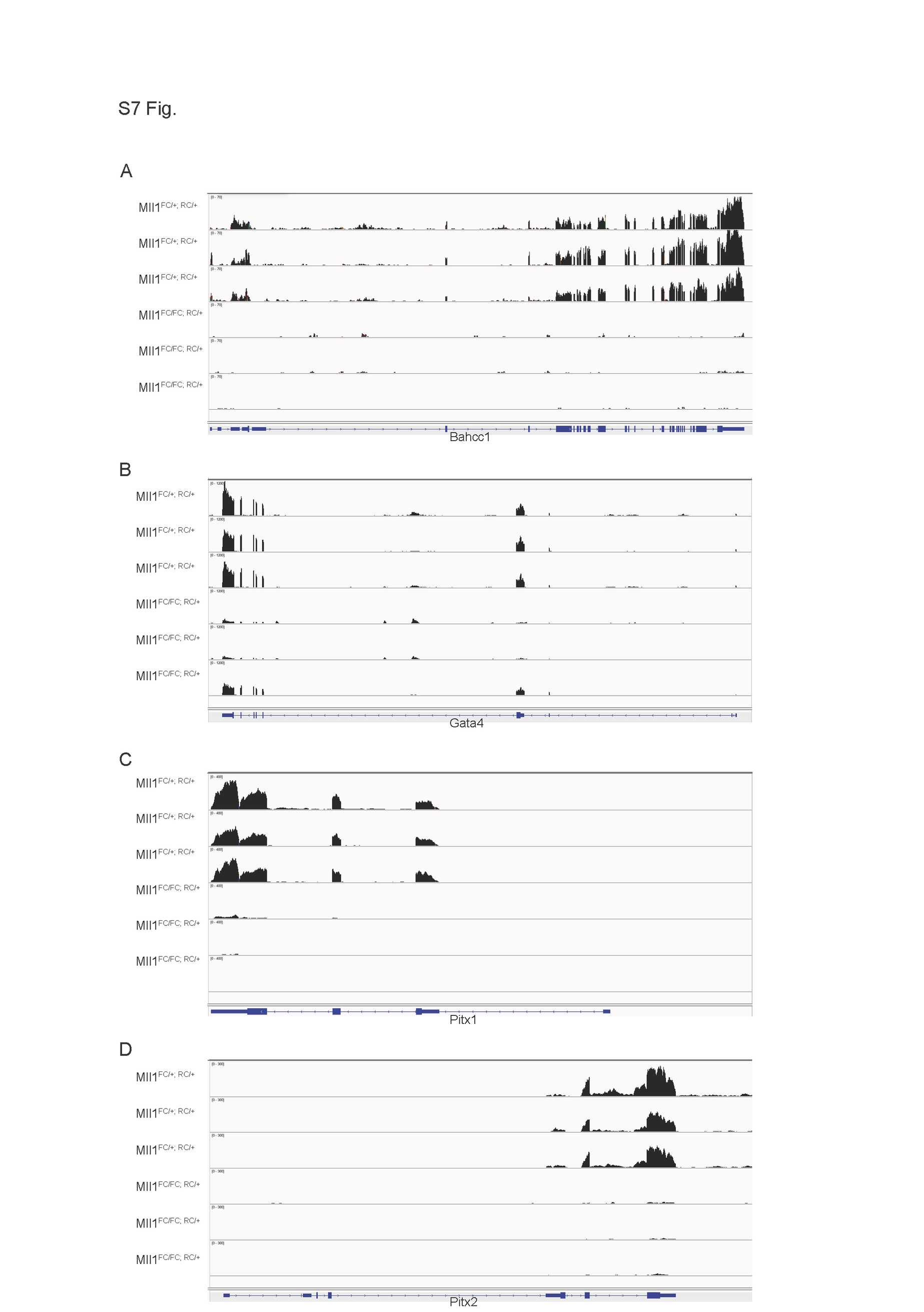

Supplement: S7 Fig — RNA-seq tracks showing the read coverage of different gene loci derived from Mll1FC/+; RC/+ (top three rows) and Mll1FC/FC; RC/+ (bottom three rows) intestinal organoids after passage 3. Gene diagrams are depicted below. The tracks were generated with the Integrative Genomics Viewer. (TIF) [file pgen.1009250.s007.tif]
