# Supplementary material for: MLL1 is required for maintenance of intestinal stem cells
Source: PLoS Genet. 2021 Dec 3;17(12):e1009250. doi: 10.1371/journal.pgen.1009250 (PMC8641872; doi:10.1371/journal.pgen.1009250)
Supplement: S1 Table — (PDF) [file pgen.1009250.s008.pdf]

Table S1. **Embryonic lethality of *Mll1*$^{A/A}$ embryos**

***Mll1*$^{A/+}$ x *Mll1*$^{A/+}$**

Age	+/+		A/+		A/A		Resorbed	Total (litters)
	Live (%)	Dead (%)	Live (%)	Dead (%)	Live (%)	Dead (%)		
Weaned	90 (35.7)	0 (0)	162 (64.3)	0 (0)	0 (0)	0 (0)$^{\#}$	0	252 (41)
**E16.5**	1 (12.5)	2 (25)	5 (62.5)	0 (0)	0 (0)	0 (0)	1	8 (1)
**E13.5**	18 (25.0)	0 (0)	44 (61.1)	0 (0)	0 (0)	10 (13.9)$^{\#}$	1	72 (8)
**E12.5**	34 (31.5)	0 (0)	53 (49.1)	0 (0)	8 (7.4)	13 (12.0)$^{\#}$	7	108 (13)
**E11.5**	32 (22.2)	0 (0)	78 (54.2)	3 (2.1)	27 (18.8)	4 (2.8)	14	144 (18)
**E10.5**	28 (22.0)	1 (0.8)	64 (50.4)	2 (1.6)	29 (22.8)	3 (2.4)	5	127 (17)
**E.9.5**	19 (21.1)	2 (2.2)	37 (41.1)	2 (2.2)	28 (31.1)	2 (2.2)	1	90 (11)
**E8.5**	0 (0)	0 (0)	1 (33.3)	0 (0)	2 (66.6)	0 (0)	0	3 (1)

# p<0.005; χ^2 test
