# Supplementary material for: MLL1 is required for maintenance of intestinal stem cells
Source: PLoS Genet. 2021 Dec 3;17(12):e1009250. doi: 10.1371/journal.pgen.1009250 (PMC8641872; doi:10.1371/journal.pgen.1009250)
Supplement: S2 Table — (PDF) [file pgen.1009250.s009.pdf]

Table S2. Primers for genotyping, qRT-PCR and ChIP-qPCR

Primer pairs	Sequence (5’ – 3’)	Product size (bp)
	**Genotyping of Cre**	
19Cre se	GCCTGCATTACCGGTCGATGCAA	726
20Cre as	GTGGCAGATGGCGCGGCAACAC	
	**Genotyping of Mll1^{A} allele**	
Mll1 loxP1	GAAGAAGTCAGAGTGCGAAGC	296 (A), 251 (wt)
Mll1 loxP2	GTAGAAACCTACTTCCCATGCC	
	**Genotyping of Mll1^{FC} allele**	
Mll1 flp se	GAGGTAAGGAGAGTTTTTGCT	1084 (F)<br>933 (wt)<br>186 (FC)
Mll1 loxP2	GTAGAAACCTACTTCCCATGCC	
	**qRT-PCR**	
Rpl19 se	CTGATCAAGGATGGGCTGATC	147
Rpl19 as	CTTCTCAGGCATCCGAGCATT	
Pitx2 se	GAGAAACCGCTACCCAGACAT	153
Pitx2 as	AAGCCATTCTTGCACAGCTCG	
Foxa1 se	GCAACGACTGGAACAGCTACT	130
Foxa1 as	GTGGTCATGGTGTTCATGGTC	
Jaml se	GGAGACGACAGAGAGAGGAAT	118
Jaml as	CTTGGATCTGACTGAAGAGCG	
	**ChIP-qPCR**	
Chr9 se	TGGCAGCTTGTGTTGTTAGA	249
Chr9 as	CCACTATGGTGTCAGGAAGG	
Bahcc1 se	TCTGGATGCCACTCGTGTTGG	119
Bahcc1 as	TCCATGGTCTGCAGAGGCAAG	
Ces2g se	TGAAACGTGGTCCTCCTAGCA	103
Ces2g as	CAGGAGTCCAAAGAACACAGC	
Far1 se	CCATAGCGCTCTCAGCCAATG	120
Far1 as	CTCTCCCTTCAGCGATGGCTA	
Foxa1 se	AGGGTTGGATGGTTGTGTCGG	102
Foxa1 as	GTGTCCGCGTAGTAGCTGTTC	
Gata4 se	TGAGCGAGTTGGGCCTCTCCT	136
Gata4 as	AACTAGAATGCGGGTGTGCGG	

Hspa8 se	CTGCGGACTGGATAAAAGCCG	110
Hspa8 as	AGGTGGTGCCGAGATCAATGC	
Jaml se	CCTGTTTCTGTTTCCGCAGCC	142
Jaml as	TGCCCAGGAAGAACAGTCCAC	
Nolz1 se	GACAAGCAGCGACACACATCG	130
Nolz1 as	GGCTAGAAGAGCAGGACAGAC	
Onecut2 se	AAGGCTGCCTACACCGCCTAT	98
Onecut2 as	CCGTGCAAAGTGCCCAGACTT	
Pitx1 se	CGCCTTCAAGGGAGGCATGAG	148
Pitx1 as	GGACGATTCGCTGGCGGAGTT	
Pitx2 se	CTGGCTCTTTCAAGTCTCGGC	135
Pitx2 as	TGCAGTTCATGGACGAGGGAG	
