# Supplementary material for: MLL1 is required for maintenance of intestinal stem cells
Source: PLoS Genet. 2021 Dec 3;17(12):e1009250. doi: 10.1371/journal.pgen.1009250 (PMC8641872; doi:10.1371/journal.pgen.1009250)
Supplement: S3 Table — (PDF) [file pgen.1009250.s010.pdf]

Table S3. Antibodies for immunohistochemistry staining and Western blot

**Primary antibodies**	**Company, Cat. Number**	**Dilution**
Rabbit anti-Mll1	Bethyl, A300-086A	1:2000
Rabbit anti-Ki67	Leica, NCL-Ki67p	1:2000
Rabbit anti-Ki67	Thermo Scientific, RM-9106	1:400
Rabbit anti-Olfm4	Cell Signaling, 39141S	1:400
Rabbit anti-Lysozyme	Dako, A0099	1:5000
Rabbit anti-CLCA3	Abcam, ab46512	1:8000
Rabbit anti-Chromogranin A	Immunostar, 20085	1:3000
Rabbit anti-Sox9	Millipore, AB5535	1:5000
Rabbit anti-β-catenin	Santa Cruz Biotechnology, sc-1496	1:100
Rabbit anti-H3K4me1	Diagenode, C15410037	1:1500
Rabbit anti-H3K4me2	Diagenode, pAb-035-050	1:1500
Rabbit anti-H3K4me3	Abcam, ab8580	1:600
Anti-BrdU (Biotin)	Abcam, ab2284	1:300
Rabbit anti-Gata4	Santa Cruz Biotechnology, sc-9053	1:1000
Rabbit anti-Gapdh	Biorbyt, orb323277	1:2000
Rabbit anti-Jaml	Abcam, ab183714	1:1000
**Secondary antibody**	**Company, Cat. Number**	**Dilution**
Biotinylated goat anti-Rabbit IgG	Vector, BA-1000	1:500
Goat anti-Rabbit IgG-HRP	Thermo Scientific, 31460	1:20000
