# Supplementary material for: MLL1 is required for maintenance of intestinal stem cells
Source: PLoS Genet. 2021 Dec 3;17(12):e1009250. doi: 10.1371/journal.pgen.1009250 (PMC8641872; doi:10.1371/journal.pgen.1009250)
Supplement: Attachment — Submitted filename: Goveas cover letter + response to reviewers.pdf [file pgen.1009250.s013.pdf]

**TECHNISCHE UNIVERSITÄT DRESDEN**

**Biotec**hnologisches Zentrum

biotec
Biotechnology Center TU Dresden

Dr. Andrea Kranz
Prof. A. Francis Stewart
Genomics Department
Center for Molecular and Cellular Bioengineering
Tatzberg 47/49
D-01307 Dresden

Contact: Juliane Wunderlich
Telephone: +49 (0)351 463-40130
Email: juliane.wunderlich@tu-dresden.de
andrea.kranz@tu-dresden.de
francis.stewart@tu-dresden.de
Date: 2021-10-22

**The Editors**
**PLoS Genetics**
**Dr. Tom Milne**

Dear PLoS Genetics editors, dear Tom,

We submit our revised manuscript "MLL1 is required for maintenance of intestinal stem cells" (PGENETICS-D-20-01516). Thank you for your constructive response of 24.06.2021 (copied below) and the insights of the reviewers. As detailed below, we have reason to thank reviewers 2 and 4 for requesting further inspection of our conclusions regarding JAML.

Dear Dr Kranz,

Thank you very much for submitting your Research Article entitled 'MLL1 is required for maintenance of intestinal stem cells and the expression of the cell adhesion molecule JAML' to PLOS Genetics.

The manuscript was fully evaluated at the editorial level and by independent peer reviewers. We have now assessed the reports from two of the original reviewers. Unfortunately, reviewer 1 was not available, but was able to suggest an alternative reviewer in their place. The new reviewer was overall positive about the paper and felt that many of the original concerns were fully addressed. However, this reviewer also raised an additional concern, that the expression changes of *Jaml* could be an artefact of inserting a cassette into the *Mll* gene (the reviewer also references the possibility of mouse strain specific issues as discussed in Mueller et al PMID: 32901003). They highlight the much larger downregulation of the *Jaml* gene compared to other loci as a point of concern, as well as the lack of H3K4me3 changes at *Jaml* (also a concern for reviewer 2). This possibility needs to be carefully considered and addressed in a carefully written response. Further analysis or additional data may be needed. Any other issues raised by the reviewers do not need to be considered in the response. Based on the reviews, we will not be able to accept this version of the manuscript, but we would be willing to review a revised version. We cannot, of course, promise publication at that time.

Should you decide to revise the manuscript for further consideration here, your revisions should address the specific points made by each reviewer. We will also require a detailed list

of your responses to the review comments and a description of the changes you have made in the manuscript.

If you decide to revise the manuscript for further consideration at PLOS Genetics, please aim to resubmit within the next 60 days, unless it will take extra time to address the concerns of the reviewers, in which case we would appreciate an expected resubmission date by email to plosgenetics@plos.org.

If present, accompanying reviewer attachments are included with this email; please notify the journal office if any appear to be missing. They will also be available for download from the link below. You can use this link to log into the system when you are ready to submit a revised version, having first consulted our Submission Checklist.

To enhance the reproducibility of your results, we recommend that you deposit your laboratory protocols in protocols.io, where a protocol can be assigned its own identifier (DOI) such that it can be cited independently in the future. Additionally, PLOS ONE offers an option to publish peer-reviewed clinical study protocols. Read more information on sharing protocols at https://plos.org/protocols?utm_medium=editorial-email&utm_source=authorletters&utm_campaign=protocols

Please be aware that our data availability policy requires that all numerical data underlying graphs or summary statistics are included with the submission, and you will need to provide this upon resubmission if not already present. In addition, we do not permit the inclusion of phrases such as "data not shown" or "unpublished results" in manuscripts. All points should be backed up by data provided with the submission.

While revising your submission, please upload your figure files to the Preflight Analysis and Conversion Engine (PACE) digital diagnostic tool.  PACE helps ensure that figures meet PLOS requirements. To use PACE, you must first register as a user. Then, login and navigate to the UPLOAD tab, where you will find detailed instructions on how to use the tool. If you encounter any issues or have any questions when using PACE, please email us at figures@plos.org.

PLOS has incorporated Similarity Check, powered by iThenticate, into its journal-wide submission system in order to screen submitted content for originality before publication. Each PLOS journal undertakes screening on a proportion of submitted articles. You will be contacted if needed following the screening process.

To resubmit, use the link below and 'Revise Submission' in the 'Submissions Needing Revision' folder.

We are sorry that we cannot be more positive about your manuscript at this stage. Please do not hesitate to contact us if you have any concerns or questions.

Yours sincerely,

Tom Milne

Thanks to reviewers 2 and 4 for requesting further inspection of our conclusions regarding JAML. To obtain the best evidence, we evaluated JAML expression by Western blot analysis. Using three commercially available antibodies (two of which worked), we realized that JAML expression was unaltered by the loss of MLL1 in organoids (Figure 1 below).

This unexpected conclusion contradicted the dramatic reduction of *Jaml* mRNA expression after *Mll1* conditional mutagenesis reported by the RNA-seq studies, which had been confirmed by *Jaml* qRT-PCR analysis. In unravelling this contradiction, we realized that several independent pieces of evidence had together been collectively misleading.

First, our RNA-seq analyses, which were based on two time points after loss of MLL1 from intestinal stem cells (ISCs) isolated by FACS and two time points after conditional mutagenesis in intestinal organoids, assigned *Jaml* as the most downregulated mRNA (ISCs) and the fifth most downregulated (P1 organoids). This strongly resonated with the listing of *Jaml* as an ISC signature mRNA by two independent studies (Munoz J et al., EMBO J 2012: Haber AL et al., Nature 2017).

Second, we confirmed the downregulated *Jaml* RNA-seq result using a qRT-PCR *Jaml* primer pair from the last two *Jaml* exons. Because the last exon of eukaryotic genes is the most conserved, we routinely choose our qRT-PCR primer pairs from the last two exons.

Third, a literature search found a PhD thesis from Hans Clevers' lab that (i) showed intestinal *Jaml* expression exclusively in ISCs *in situ*; and (ii) used conditional mutagenesis to establish the requirement for *Jaml* in ISC maintenance with a similar phenotype as the conditional knockout of *Mll1* (Tetteh PW. Plasticity of intestinal epithelial cells in regeneration and cancer. Utrecht University; 2015). In particular, after conditional mutagenesis, both *Mll1* and *Jaml* KO phenotypes include stem cell defects and a loss of proliferation.

Based on this evidence we confidently drew the conclusion that MLL1 regulates *Jaml* expression, and both proteins are required for ISC maintenance.

Since receiving the comments from reviewers 2 and 4, multiple JAML Western blots and several new *Jaml* qRT-PCR primer pairs across the gene overthrew this confidence. We realized that lncRNAs overlapping the last two *Jaml* exons are the transcripts that are dramatically downregulated upon MLL1 ablation (Figure 2 below). In common with many labs, we routinely ignore the eclectic partial transcripts annotated (by automatic data processing) onto the mammalian genome browsers. Due to our recent re-analysis, we now realize that the downregulated transcript is not *Jaml* but obscure lncRNAs that overlap the last two *Jaml* exons (Figure 2 below). Our original choice of PCR primer pairs from the last two exons was made long before we had any notion of the overlapping lncRNAs and was bad luck. In the absence of MLL1, levels of *Jaml* mRNA and protein are unaffected, proving that these lncRNAs do not regulate JAML expression. What do these new lncRNA conclusions mean? Like many lncRNA phenomena, we have no worthwhile explanation.

The manuscript has been adjusted to remove the prominence of JAML and now includes a brief comment about the overlapping lncRNAs.

Because Sashimi and coverage plots were vital in revealing the mistaken assignment to *Jaml* and the identity of the lncRNA, we now include a selection of these plots for key mRNAs in the Supplement. The manuscript has also been improved in several other ways, including Western blot confirmation of the relationship between MLL1 and GATA4 (new Fig. 4G). Our efforts to include Western blot data for the other prominent transcription factors have been frustrated by the poor quality of available antibodies. The revised manuscript also includes a modest number of textual alterations to improve expression.

Below we respond point-by-point to the reviewer's comments.

Reviewer #2: The revised manuscript further strengthened the major conclusion that Mll1 is required for intestinal stem cell maintenance and expression of certain key transcriptional regulators and JAML. The phenotypic and gene expression analyses are rigorous. The conclusions are significant and improve our understanding of the critical role of Mll1 in somatic stem cell biology.

The mechanisms by which Mll1 regulate these few genes are unknown. Mll1 ChIP is indeed technically challenging. The newly added H3K4me3 ChIP-qPCR data on the affected genes are helpful. However, it is somewhat disappointing that the authors did not pursue a more thorough assay to find out whether H3K4me3 at JAML is truly affected, since JAML is claimed to be the single most Mll1-dependent gene in these cells (and in the title!) and may be functionally relevant for ISC regulation by Mll1. It is surprising to see almost no H3K4me3 signal at the JAML promoter, since this gene seems to be expressed at a pretty high level and there is a well-established correlation of gene expression level and H3K4me3 level at transcription start site. The gene may have multiple TSSs and the choice could be tissue specific. In fact, H3K4me3 peak is probably the most reliable mark for an active true TSS. The primers most likely have missed the true TSS in this cell type. It matters because data presented in the current manner may (mis-)lead the readers to think that Mll1 must act on its most important target gene in intestinal stem cells through a methylation-independent way. H3K4me3 ChIP-seq is really not much more work than ChIP-qPCR, and would show the genome-wide impact of losing Mll1. As the H3K4 methylation-dependent and -independent activities of Mll1 remain poorly understood, the integrative analysis of the effects of Mll1 loss on genome-wide methylation and expression may provide some insight that could be interesting and useful to the epigenetics research field. Even if ChIP-seq is not done, the authors should at least test H3K4me3 by qPCR using primers at all alternative TSSs in updated database. There are also large number of H3K4me3 ChIP-seq data from mouse cells (especially ES cells) in databases that could aid in primer selection, though TSS in ISCs could be different. In fact, using publicly available mouse ES cell H3K4me3 ChIP-seq data show a prominent peak near the center of this gene (an alternative TSS), not at the annotated TSS detected by the primers used in this manuscript. Anyway, simply using one primer pair

is not convincing (and could be misleading) for this target that is claimed to be the most important Mll1 target in ISCs.

We thank the reviewer for these thoughtful comments. As described above, JAML is no longer relevant for the MLL1 phenotype and the loss of H3K4me3 indeed serves as confirmation of the MLL1 target genes.

Reviewer #3: In my report, I have kept at minimum my suggestions (only 4 points), in order to allow the authors to focus on those important aspects.
Yet, none of the criticisms I made were addressed.
The suggestions I made are important and should addressed before publication.

Please recall that previous editorial guidance excused the need to respond to the sweeping suggestions of reviewer 3 for considerably more data (including mass spectrometry, rescue experiments …). This guidance appears to be reiterated in the letter above "Any other issues raised by the reviewers do not need to be considered in the response." Please let us know if we are mistaken.

Reviewer #4: Goveas et al. have shown a requirement of the H3K4 methyltransferase Mll1(Kmt2a) for small intestine homeostasis, adding up a new stem cell compartment to the Mll1 repertoire. They have used an elegant conditional mouse mutagenesis strategy to delete Kmt2a in the whole body except HSCs (Rosa-CreER + WT BM transplantation) or specifically in the intestinal compartment (Villin-CreER). In both strategies they have observed a rapid decline of the mouse health with collapse of the intestinal function.
In this revised manuscript, the authors addressed successfully some of the reviewer's concerns (note: I have not reviewed the first submission). However, there is a key aspect that has been missed and I believe needs to be clearly addressed:
Gene expression analysis of LGR5+ SCs did show modest fold changes in a small number of transcripts with only one transcript (Jaml) being significantly (34fold) down-regulated. Additional transcriptome analysis of P1 organoids has uncovered Mcam as the most down-regulated transcript.
Jaml (Amica1) and Mcam are neighbouring genes to Kmt2a. These 2 genes are by far the most down-regulated genes in RNAseq in Lgr5+_SC and P1 organoids. This is a potential source of artefacts since the transgenesis process has introduced a cassette that could disrupt expression of adjacent genes or even introduce mouse strain specific germline expression bias similar to this reference (Mueller, Lange et al. 2020). To strengthen this concern the authors have not identified many other significantly up- or down-regulated genes with more than 1fold change (log2) in ISCs. Moreover, Mll1 deletion did not cause a decrease on H3K4me on the Jaml promoter (Fig4H). The appropriate control mice would be a non-induced Mll1FC/FC to buffer any potential effects of transgenesis, in addition to Mll1FC/+ used by the authors. This is a key control since the authors base a lot of the discussion in the potential effects of Jaml down-regulation in the phenotype observed.
I understand that re-doing these experiments in the mice is time-consuming but they would be important to the wider community using this Mll1 mouse model in future experiments. Organoid cultures could be used as a quicker/cheaper alternative. Gene editing or RNAi on Mll1 should also be used to exclude any effects of the targeting allele/Cre recombination on Jaml expression (or other expressed neighbour genes such as Mcam).

Again we thank the reviewer for a very careful observation - that the *Jaml* locus is 950 kb upstream of *Mll1.* The fact that JAML expression is unaffected by loss of MLL1 diminishes the primary concern about cis-acting problems caused by the targeting of *Mll1*. Nevertheless, to examine this possibility, we

(i) performed Southern blots to check the targeting event using tissue from the mice (previously the Southern blots were performed on the targeted ES cells). These repeated Southern blots confirm the accuracy of the targeting and are now displayed in the Supplement in place of the previous Southern blots (Fig. S1C).

(ii) looked at genes in the 950 kb between *Mll1* and *Jaml*. In our organoid RNA-seq, four genes in this interval are expressed and none are affected by loss of MLL1. Values are average reads -

| | | | | | |
|---|---|---|---|---|---|
| Atp5l | 1042 (C) | 1132 (KO); | Ube4a | 3680 (C) | 3310 (KO) |
| Mpzl2 | 4165 (C) | 4137 (KO); | Mpzl3 | 2123(C) | 2144 (KO). |

(iii) looked at *Mcam* in more detail. After loss of MLL1, *Mcam*, which is 700 kb downstream of *Mll1*, was the most downregulated mRNA in the P1 organoid experiment. However, *Mcam* was not downregulated in the ISC transcriptome data, arguing against an impact of targeting on its expression.

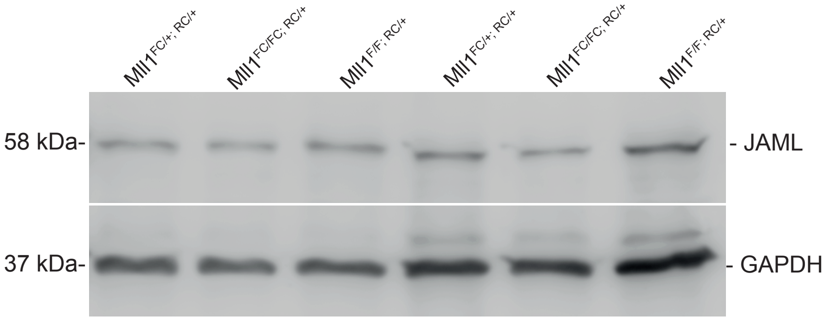


**Figure 1.** Western blot analysis shows no change in JAML protein levels in *Mll1*$^{FC/+; RC/+}$, *Mll1*$^{FC/FC; RC/+}$ and *Mll1*$^{F/F; RC/+}$ organoids (n=2). GAPDH is the loading control.

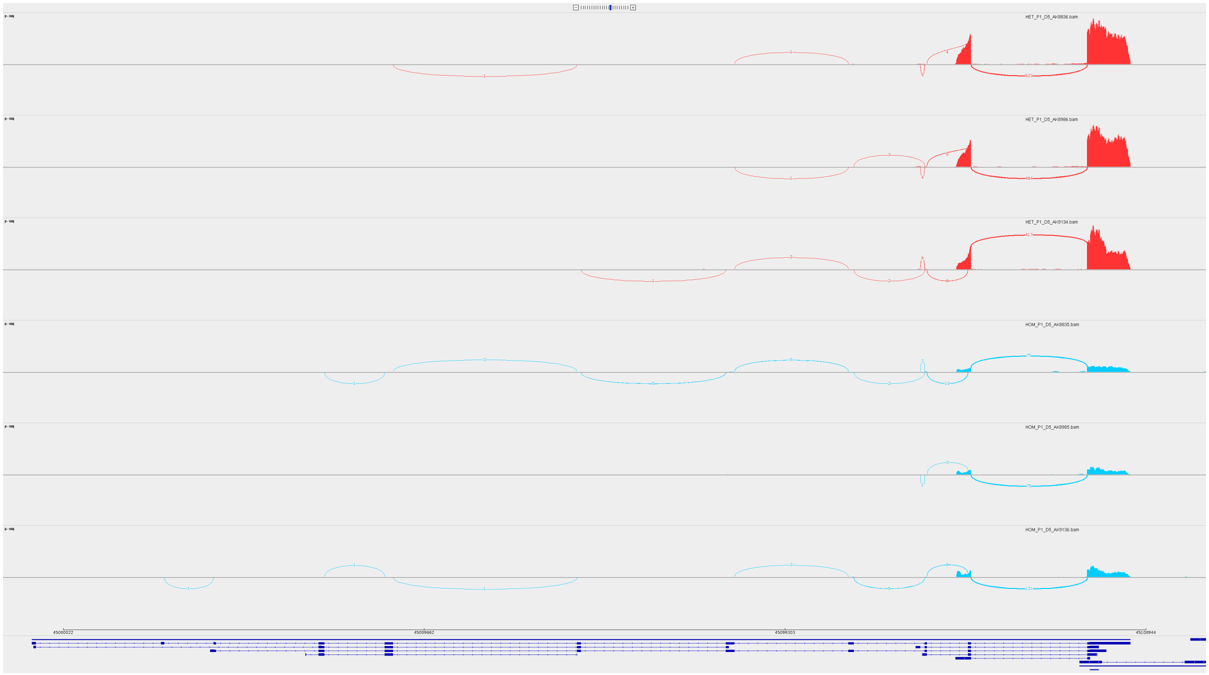

**Figure 2. Panel above**: Sashimi plot of mapped reads on the *Jaml* gene from the P1 organoid experiment. The triplicates are shown with the *Mll1*$^{FC/+; RC/+}$ above in red and the *Mll1*$^{FC/FC; RC/+}$ below in blue. At the bottom is the gene diagram showing *Jaml* exons in dark blue. Transcription is from left to right. Underneath the 3' end are lncRNA diagrams – see enlargement below.
**Panel below**: Coverage plot showing read species at the 3'end of the *Jaml* gene with the exon diagram below. The 3' end of the *Jaml* gene is on the first and third lines of the diagram (different mRNA isoforms). The second line shows an annotated RNA transcript (termed '207' on the ENSEMBL browser). The fourth and fifth lines illustrate two other RNA transcripts (termed '203' and '202' on the ENSEMBL browser). The sixth and seventh lines show lncRNAs from the other strand (i.e. transcripts from right to left). The existence of the lncRNAs (202, 203, 207) was confirmed using 5' PCR primers unique to the lncRNAs from the indicated exons (red arrows) with a 3' primer from the last *Jaml* exon. Inspection of the Sashimi plot (above) indicates that most of the lncRNAs that overlap the last two *Jaml* exons include the entire *Jaml* 3' UTR, which is not included in the diagrams of lines 2, 4 and 5 or 202, 203 and 207 on the ENSEMBL browser.

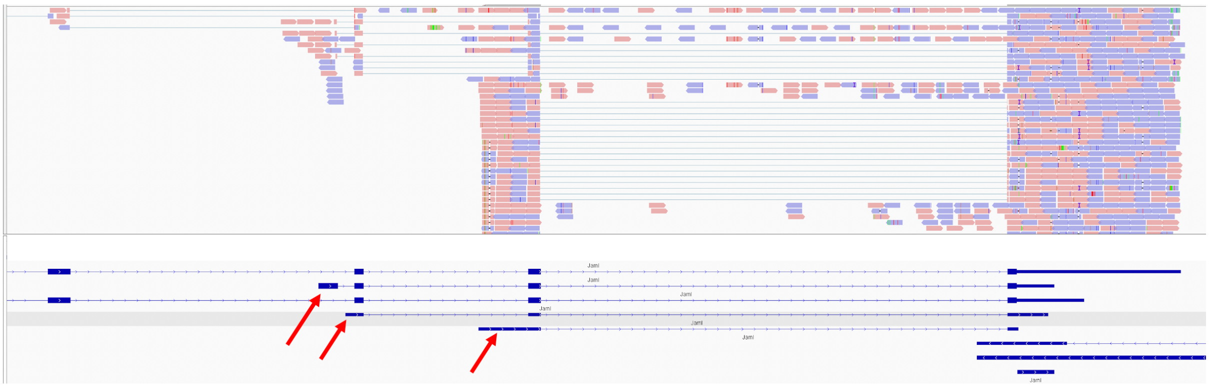

We are exceptionally grateful to the reviewing process that helped us identify a substantial error. We hope that the reviewers and editors agree that the manuscript now meets the high standards required for publication in your pages. We remain willing to incorporate further advice and look forward to your response.

Regards,

Andrea Kranz

Francis Stewart
