# Supplementary material for: MLL1 is required for maintenance of intestinal stem cells
Source: PLoS Genet. 2021 Dec 3;17(12):e1009250. doi: 10.1371/journal.pgen.1009250 (PMC8641872; doi:10.1371/journal.pgen.1009250)
Supplement: Attachment — Submitted filename: Goveas point by point response.doc [file pgen.1009250.s012.doc]

Point-by-point response

**Reviewer #1:**

The submission by Goveas builds on the interest of the group on the role of Mll1 (actually Kmt2a) an important methyltransferase that methylates Histone 3, lysine 4 residues (H3K4) to epigenetically regulate gene expression. Here the authors investigate the role of Mll1 in maintaining intestinal stem cells. Their interest arises from other tissue stem cells systems in which Mll1 has a central role including, most notably, haematopoietic but also neural and muscle satellite stem cells. In establishing Mll1 as a master regulator of stem cells the authors wish, at least in part, to perform a comparative analysis across different tissues and identify common themes or mechanism of action.

The work described does persuade that Mll1 has an important role in maintaining intestinal stem cells. However the authors account is overly simplified. They approach the topic in a blinkered way that provides an incomplete account of the work described to provide a narrative that is phenomenological with little insight into mechanism.

1. The gene name is now Kmt2a, should this not be adopted throughout?

Although the official gene name is *Kmt2a*, there are many reasons to keep using the earlier and more widely known name of this very famous gene, *Mll1*, which is preferred by almost everybody in the field.

Personal comment on nomenclature from AFS is attached at the end of this response.

2. The mouse used is described as though for the first time. Yet different descriptions of the floxed allele are confusing:
Line 160 refers to a likely null allele while line 167 refers to the same floxed allele and both claims are supported by ref 6 of the manuscript. Accepting the latter: why is much of the characterisation of the parental ‘A’ allele line needed if the floxed line derived from it by Flp recombination is validated?
The *Mll* alleles described here are not the same as Chen et al., 2017, rather both our A/A and FC/FC versions deliver the same phenotype as each other and the *Mll1* allele published in Chen et al., 2017, which now establishes all 3 as the null. This is important because the first three “knock-out” alleles of *Mll1* (Yu et al., 1998, Yagi et al., 1998, Ayton et al., 2001) delivered very different phenotypes and the issue of the *Mll1* null remains unresolved until now. Although peripheral to the intestinal theme, we briefly include these considerations of the various *Mll1* alleles for clarification in the specialist field. We have not previously published phenotypic descriptions of either our *A/A* or *FC/FC* alleles.

The nomenclature used to describe the different recombined state of the modified allele is confusing. A floxed allele would often be presented as the gene name with ‘f/f’ or ‘f/wt’ in superscript. Then recombination would be designated by ‘delta’or ‘rec’. Instead here the authors use F and C to designate that the allele has already undergone Flp and/or Cre recombination.
The Flp recombination being constantly referred to implies its use in the experiments described but in fact this done once from the parental ‘A’ allele to generate what is essentially floxed conditional Mlll1 KO.

The nomenclature for allele conversion by site specific recombinases remains confusing, particularly within conditional mutagenesis. After site specific recombination by FLP or Cre recombinases, two conventions are in practice. Either the A, B, C, D convention partly adopted by International Knock out consortium (A = targeted allele, B = FLPed, C = Creed, D = both) (Skarnes et al., 2011) or the A, F, C convention that makes more intuitive sense (A = targeted allele, F = FLPed, C = Creed, FC = both) and can easily accommodate other recombinases like Dre (D). We have always used and continue to use the AFC nomenclature.

3. The early part of the manuscript documents an intestinal phenotype for Mll1 deletion by first reconstituting the bone marrow after lethal dose irradiation with wild type cells and then mediating Mll1 deletion body wide with tamoxifen. This is an inelegant solution, the gut is probably the next most sensitive tissue to irradiation so the phenotype is being described in an already damaged tissue system. The mice go down within 14 days or so with diarrhoea. The intestinal characterisation lacks key details of time post induction that analyses were done, whether the mice analysed were symptomatic or not and lacks definitive evidence that failure of the epithelial lining is the principle cause of the phenotype (as opposed, say, to compromised immune monitoring due the BM reconstitution and irradiation).

As stated before and now more explicitly clear due to the inclusion of new Supplementary Figure 2A, the intestinal phenotype and onset of diarrhea after loss of MLL1 is identical between the irradiated (Fig. 1D) and non-irradiated mice (Fig. S2A). The irradiated, bone marrow transplanted, mice did not suffer from diarrhea or weight loss, which are usually symptoms of radiation induced intestinal injury, during the phase of stable engraftment of the transplant and the following recovery phase. Mice had more than three months to recover before tamoxifen gavage to induce conditional mutagenesis. Therefore potential irradiation damage to the intestine had no impact on the experiment.

3. Why is colon not included in this or subsequent analyses? Was there a phenotype?

We focused on the small intestine because intestinal tumourigenesis in mice arises in the small intestine rather than the large as in humans (Janssens et al., 2002). This primary difference between mice and human intestine has been recognized for some time. We have stained colonic sections with the genotype *Mll1^FC/+; RC/+^* (n=3) and *Mll1^FC/FC; RC/+^* (n=3) for GOB5, SOX9 and Ki67 but did not observe dramatic effects (see attached Fig. 1). We did not further pursue the colon and are unable to present a publishable analysis.

Figure 1. Antibody stainings of SOX9, Ki67 and GOB5 on *Mll1^FC/+; RC/+^* (n=3) and *Mll1^FC/FC; RC/+^* (n=3) colonic sections (A, B, C). Scale bars are 100 μm.

4. The subsequent validation with the more straightforward intestinal deletion of Mll1 using VillinCreER mice only partially reassures because the analysis is treated as confirmatory and is superficial.

What is the outcome of Mll1 deletion; can mice be maintained long term? Why is a survival analysis not performed? When was weight loss observed? When post-induction were the analyses shown performed?

We thank the reviewer for asking for a more detailed presentation of the Villin-CreER^T2^ experiments. Previously we favoured brevity and a focused presentation of the key results. However, a more detailed presentation of additional aspects of this conditional experiment is informative and also addresses a further point made by the reviewer regarding the temporal order of events. The fuller analysis is now presented in the manuscript line 221-246 along with revised Figure 3 and new Supplementary Figure 2G.

4. Overall there does seem to be a phenotypic downregulation of stem cell and proliferative markers and a skew in lineage composition towards goblet cells following Mll1 deletion. Assigning significance to these observations requires a better understanding of the temporal sequence leading to these changes, how they impact on stem cell function (loss of marker doesn’t necessarily equate with loss of function) and mouse survival.

Note: We observed loss of two stem cell markers, OLFM4 and SOX9 in *Mll1^FC/FC; Vil-Cre-ERT2/+^* mice.

Regarding the sequential order of events taking place in the intestine we now present a further aspect of the Villin-CreER^T2^ experiment. Three *Mll1^FC/FC; Vil-Cre-ERT2/+^* mice were analyzed as early as feasible (~ 7 days after the first tamoxifen gavage). In these sections, the stem cell marker, OLFM4 and the proliferation marker, Ki67, were clearly decreased. However, alcian blue staining revealed only a slight increase in the goblet cell population in *Mll1^FC/FC; Vil-Cre-ERT2/+^* intestinal sections indicating that the impact on goblet cells is later than the primary impact on ISCs. These early data are now included as new Figure 3E.

5. The authors move to the Lgr5 creER model as this allows stem cells to be directly isolated due to the GFP allele in the Lgr5 locus. There seems something of a missed opportunity here as the Lgr5CrerER mice are well known to be mosaic in their expression. The longer term phenotypically consequences of Mll1 loss could have been determined with mice remaining viable due to incomplete loss of Mll1.

Our primary phenotype in the *Mll1^FC/FC; RC/+^* and *Mll1^FC/FC; Vil-Cre-ERT2/+^* line was wasting or diarrhea and the loss of ISCs after loss of MLL1. We did not observe wasting or diarrhea when using the *Lgr5-eGFP-CreERT2* line. However further analysis of the intestine in the *Lgr5-eGFP-CreERT2* line by the Birchmeier lab (co-authors here) supports the conclusion that conditional mutagenesis by tamoxifen induction of *Lgr5-eGFP-CreERT2* is mosaic and occurs at a moderate frequency that allows replenishment by the intestinal system. These data are currently in a new Birchmeier lab manuscript that will be submitted soon.

5. Also the assumption in looking at 4 and 10 day post the last dose of tamoxifen (day 7 and 13 post first injection) is that there is no phenotypic consequence yet apparent follow Mll1 ablation. This is important, is Lgr5GFP downregulated at these times? How might that impact on the analysis that follows? Perhaps the gating strategy is not randomly sampling the stem cell pool but collecting a subpopulation of Lgr5GFP expressing cells. An in situ analysis is required here.

We were also concerned about the potential impact of ISC loss on the transcriptome analysis, which was based on sorting cells for GFP expression from the polycistronic Lgr5 transgene that expresses CreERT2. Our concerns were appeased by the excellent concordance between 4 day and 10 day downregulated mRNAs (as most clearly presented in both the previous and revised versions of Table 1). We are confident that the 4 day GFP positive population is unaffected by ISC loss however we did see a decrease in *Mll1^FC/FC; Lgr5-Cre-ERT2/+^* stem and Paneth cells numbers 10 days after tamoxifen induction (attached Fig. 2). Please note; PCR genotyping confirmed deletion of *Mll1* solely in the stem cell compartment (Fig. S3C).

Figure 2. Number of sorted stem and Paneth cells, 4 and 10 days after tamoxifen induction was completed.

We sorted Lgr5-GFP^hi^ stem and Paneth cells from *Lgr5-eGFP-CreERT2* mice not treated with tamoxifen The fidelity of the sorted cells was confirmed by qRT-PCR expression analysis using stem and Paneth cell markers. The stem cell markers, *Olfm4* and *Ascl2* were strongly enriched in the sorted stem cells (attached Fig. 3A). Transcripts from the Paneth cell markers, Lysozyme and *Mmp7* were strongly enriched in the Paneth cell fraction (attached Fig. 3B).

Additionally, we seeded these sorted Lgr5-GFP^hi^ stem and Paneth cells into matrigel with organoid medium. Paneth cells formed larger aggregates as compared to stem cells and slowly died (data not shown). Only the Lgr5-GFP^hi^ stem cell fraction showed GFP expression and were able to grow into organoids that were apparently the same as those derived from whole crypts (attached Fig. 3C).

Figure 3. qRT-PCR was performed for stem cell markers, *Olfm4* and *Ascl2* (A) and Paneth cell markers, Lysozyme and *Mmp7* (B) on sorted stem cells (SC) and Paneth cells (PC). Lgr5-GFP^hi^ sorted single cells were assessed for their growth potential in organoid culture conditions. (C) An example of a successfully growing organoid arising from a single GFP^hi^ cell. Red arrows point to GFP^+^ stem cells. Scale bar is 100 μm.

5. The mechanistic insights come entirely from transcriptional changes following Mll1 ablation in Lgr5 stem cells. JAML is a potentially interesting mediator and a number of transcription factors are identified. As the authors point out deletion of Jaml in the intestine replicates much of the phenotype they describe for Mll1. However, some reassurance is needed that these candidate mediators, especially the transcription factors not known as Mll1 target genes are not altered as a secondary consequence of a developing phenotype (eg fewer enterocytes, elevated goblet cells).

We chose the 4 day time point to observe the earliest transcriptional effects of *Mll1* deletion and thereby improve the confidence in assigning direct MLL1 targets and deducing mechanism. The new ChIP results now included in line 316-324 and 684-702 along with Figure 4H do add some reassurance to the conclusion that these transcription factors are direct MLL1 targets. The downregulated genes obtained from the RNA-seq data were confirmed with qRT-PCR on 4 day sorted ISCs (Fig S5C).

Furthermore, the concordance with the new organoid transcriptome analysis also adds further reassurance.

6. The organoid experiments might have offered a good functional test of impaired self renewal capacity of stem cells following Mll1 ablation. However, the phenotype here did not reflect that in vivo. The cystic structures formed seem reminiscent of the spheroids that form when levels of wnt signalling are elevated.

We have included RNA-seq data on *Mll1^FC/+; RC/+^* and *Mll1^FC/FC; RC/+^* organoid cultures in the main manuscript. Significant biological processes associated with the up-regulated mRNAs were ‘regulation of cell proliferation’, ‘cell adhesion’, ‘epithelium development' and ‘Wnt-signaling pathway’. This is in agreement with the formation of spheres. Please refer to line 342-382 and 654-675 along with updated Table 1, Figure 6 and new Supplementary Figure 6. The difference in phenotype *in vivo* and *in vitro* might be due to signaling from various mesenchymal and connective tissue cells *in vivo* whereas organoids grown in culture lack these signals.

6. The authors could try seeding Mll1 deficient cells or crypts and see if they have impaired seeding efficiencies.

We established organoids after tamoxifen induction *in vivo*. Crypts were harvested from ten-week old *Mll1^FC/+; RC/+^* and *Mll1^FC/FC; RC/+^* mice seven days after tamoxifen induction was completed. The absence of MLL1 resulted in a reduced number of crypts (attached Fig. 4).

Figure 4. Mice with genotype *Mll1^F/+; RC/+^* and *Mll1^F/F; RC/+^* were induced with tamoxifen for 4 days and dissected 1 week later. Crypts were isolated from the jejunum and counted. Mean+s.d. is shown; n=3; *p<0.05, Student’s *t* test.

Further, the purified crypts were seeded to check the organoid forming capacity (attached Fig. 5). *Mll1^FC/FC; RC/+^* cultures showed a decreased number of healthy/budding organoids compared to the control (*Mll1^FC/+; RC/+^*) and increased cell death by day 5 (attached Fig. 5A and 5B). qRT-PCR revealed mRNA downregulation of *Jaml* and transcription factors such as *Foxa1*, *Gata4*, *Pitx2* and *Zfp503* in *Mll1^FC/FC; RC/+^* crypts (attached Fig. 5C).

Figure 5. (A) Differential interference contrast (DIC) images of *Mll1^FC/+;^ ^RC/+^* and *Mll1^FC/FC;^ ^RC/+^* organoids. Mice were induced with tamoxifen for 4 days and dissected 1 week later. Purified crypts were seeded to check the organoid forming capacity. At passage 0 (P0) the mutant displays increased cell death. Scale bar is 100 μm. (B) Quantification of healthy organoids, dead organoids, non-budding organoids and spheres at P0 day 5. (C) qRT-PCR was performed for selected genes on cDNA from *Mll1^FC/+; RC/+^* and *Mll1^FC/FC; RC/+^* crypts. Mean+s.d. is shown; n=3; *p<0.05, **p<0.01, Student’s *t* test.

These results are generally compatible with the other results and were not pursued further.

**Reviewer #2:**

The manuscript by Goveas used a temporally and/spatially controlled Mll1 knockout mouse model to show that Mll1 is required for maintenance of intestinal stem cells (ISCs). They first show that global deletion of Mll1 in adult mouse with wild-type hematopoietic system (by transplanted bone marrow) suffer from diarrhea and wasting, indicating functional defects in the intestinal system. Morphological, immunohistochemistry, and RNA-seq together suggest reduced ISC identity and cell proliferation, as well as increased goblet cells. Similar conclusions are confirmed by intestinal epithelium-specific KO and also using cultured organoids. They also identified a limited number of genes that altered expression levels upon Mll1 loss in ISCs. Interestingly, in addition to a number of transcription factors, a rather specific gene, JAML, was found to be particularly dependent on Mll1 in ISCs. This is similar to some of their previous studies in which they found Mll1 or Mll2 is particularly important for a few specific target genes. They also propose that Mll1 is likely a master stem cell regulator, considering its importance of a number of different somatic stem cells.

This work is original, and the methodologies are rigorous. The data are substantial for supporting the conclusions. This work expands our understanding of Mll1 as a key regulator of adult stem cells.

It is important to do co-staining of Ki67 with certain cell identity markers to show what types of cells show reduced proliferation.

We presented co-staining of Ki67 with alcian blue, which stains the mucus present in mature and immature goblet cells. Ki67 primarily stains the TA proliferative compartment and does not co-stain the goblet cells or other mature cell types.

It remains a mystery how Mll1/2 is specifically required for just a few target genes in a tissue-specific manner. It will be helpful to determine Mll1 binding and H3K4 methylation at the affected genes using relevant cells from animals or organoids. It could be argued that previous reports show Mll1 binds to nearly all active gene promoters and the H3K4 methylation activity of Mll1 does not seem to be the critical for certain physiological processes. However, this is a new system, and unaffected H3K4 methylation at the affected genes will help rule out Mll1’s methylation activity as a key mediator in maintaining these genes. If cell number is a limiting factor for ChIP, the authors may want to consider the newly developed CUT&RUN, which seems easier, requires far less number of cells, and have a few other advantages compared to ChIP. Any effort toward elucidating the direct effect will be appreciated.

We and others have published that MLL1 appears to bind almost all active gene promoters and the H3K4 methylation activity does not seem critical. The reviewer acknowledges this exasperating aspect of the H3K4 methylation system. Our extensive ChIP-seq mapping of the MLLs and SETDs has all been based on tagged proteins. The antibodies against MLL1 are particularly difficult to use in ChIP and by and large deliver indifferent outcomes. Reduced H3K4me3 is a more reliable indicator but only when a significant effect is recorded. Redundancies in the H3K4 methylation system can obscure conclusions about direct targets.

In response to the reviewers comments, we performed H3K4me3 ChIP-PCR experiments on the prominent downregulated genes. *Mll1^F/+; RC/+^* and *Mll1^F/F; RC/+^* mice were induced with tamoxifen for four days and crypts were isolated 1 week after the last tamoxifen gavage. Deletion of *Mll1* caused decreased H3K4me3 on *Pitx1*/*Pitx2*, *Gata4*, *Foxa1*, *Bahcc1*, *Zfp503* and *Onecut2* promoters, but disappointingly not on the *Jaml* promoter. These results are in line 316-324 and 684-702 along with new Figure 4H.
 **Reviewer #3:**

Methylation of histone H3 on lysine 4 (H3K4me) correlates with active promoters and enhancers. These post-translational epigenetic modifications are deposited by Set1/Trithorax histone methyltransferase enzymes, which reside in large multi-protein complexes. While, MLL3 and MLL4 are predominantly associated with the deposition of H3K4me1 at enhancers, recent data in mouse embryonic stem cells indicate that MLL2 complex is implicated primarily in catalysing the deposition of the methyl mark at ‘bivalent’ developmentally regulated promoters. With respect to MLL1, genome-wide data shown its association with a subset of active promoters/enhancer. Importantly, the mechanisms governing the occupancy of the Set1/Trithorax complexes are still poorly understood. Moreover, as mentioned above, the function of the MLL complexes has been deeply studied in (mouse) embryonic stem cells, while their role in adult stem cells is still quite unclear.
In this manuscript, Goveas and coworkers investigated the role of MLL1 in the intestinal epithelium. By a clever ligand-induced conditional mutagenesis strategy, there were able to abrogate the expression of MLL1 in the gut epithelium of young mice. With this approach, the authors conclude that MLL1 is necessary for preserving the integrity of the at the base of the crypts where the intestinal stem cells (ISCs) are located. Moreover, it seems that MLL1 also implicated in maintaining the balance between secretory and absorptive cell lineages. Indeed, genetic deletion of MLL1 triggers the down-regulation of several genes, among those Jaml, a cell adhesion molecule. The effects of MLL1 deletion were further studied in organoids generated from isolated crypts.
Overall, the data presented here are robust and interesting. The paper is well-written and easy to read. The Achilles tendon for this paper is the lack of mechanism behind the effects observed upon genetic deletion of MLL1. Without further insights into the mechanism of MLL1 regulation of ISCs I would say that this manuscript does not provide a major advance in our knowledge of MLL1 or in its role in adult stem cells biology.
I thus would suggest few lines of experiments that can help the authors to elucidate MLL’s role in ISCs.

1) It is unclear whether the enzymatic activity of MLL1 is implicated in the observed phenotype. Moreover, the enzymatic role for Set1a has been recently put under discussion (PMID: 28939616 (Christie, Shilatifard 2017 Setd1a)). The authors should perform experiments in this direction: attempting the rescue the genetic deletion using enzymatically inactive MLL1, or by similar approaches. Furthermore, the analysis of ChIPseq data for H3K4me will also help in dissecting the methyltransferase-dependent and methyltransferase-independent functions of MLL1.

Although interesting, our manuscript does not embrace the issue of methyltransferase-dependent and methyltransferase-independent functions of MLL1. As a huge protein, MLL1 has many other associated functions and functional domains and addressing a structure-function analysis of MLL1 is beyond the scope of this study.

2) The authors should identify direct versus indirect effect of MLL1 deletion by combining RNAseq with ChIPseq data.

As discussed above, ChIP results from the H3K4 system have been exasperating due to promiscuous binding and redundancy within the system. Nevertheless, our new ChIP-PCR results do suggest that several transcription factor promoters are direct targets (line 316-324 and 684-702, Fig. 4H).

3) It has been reported that MLL proteins do not activate gene expression but rather prevents a default repressed state, often related to Polycomb occupancy. Is thus PRC1 and/or PRC2 accumulated at down regulated genes? Or is their silencing due to DNA methylation?

This issue is important and will require a new dedicated project – possibly a PhD project. Previously we identified a rare target gene (*Magoh2*) whose expression in ES cells entirely depended on MLL2. Analysis *Magoh2* regulation by MLL2 supported the anti-repression scenario (Glaser et al., 2009, Ladopoulos et al., 2013). Here we identify *Jaml* as a candidate for regulation by MLL1 anti-repression.

4) Since the targeting of MLL1 to specific loci is still poorly understood, the authors could further investigate this aspect by analysing the interactome of MLL1 in ISCs or in organoids.

We thank the reviewer for these suggestions for future work. However these interactome and mass spectrometry exercises would require a large number of mice and in Germany, we probably would not get permission.

AFS comment on Kmt nomenclature.

In 2007, an email proposing name changes for the histone methyltransferases and demethylases was circulated. I was one of the recipients. My response focused on the H3K4 methyltransferases.

I objected to the proposed names of the H3K4 methyltransferases for the following reasons:

i) The name Mll1 had been agreed after substantial conflict between the labs that had independently discovered, in the early 1990s, probably the most important gene in human leukemia. In 2007, everyone in the field had only recently agreed to stop the squabble. Jay Hess, who also received the email, independently made the same point. Mll1 was, and is, a much better name, especially for clinicians.

ii) By the nomenclature rules that had been defined in the 2007 email (which defined Kmt1a as the first discovered H3K4 methyltransferase, therefore “1” and “a”), Kmt2a should be the second discovered class of histone methyltransferases, which was the H3K4 methyltransferase Set1 from yeast, published by my lab, not Mll1, which was indirectly proven to be an H3K4 methyltransferase a year later. However, Set 1 and it’s mammalian homologues, Setd1a and Setd1b were assigned Kmt2f and Kmt2g, which made no sense at all.

iii) A gene termed Mll5 because it had a SET domain and a PHD finger (like its’ yeast homologue, Set3, which was first characterized by my lab), was termed Kmt2e. This also made no sense, in part because it could not be a methyltransferase. In 2007, my email response pointed out that the known SET domain essential conserved amino acids for methyltransferase activity were missing and also, Mll5 in mammals had a closely conserved sister gene that was not included in the nomenclature. (Notably, in 2009 the serial fraudster, Kato of Tokyo University published fraudulent data in Nature supposedly proving that Mll5 was an H3K4 methyltransferase, shortly before his exposure).

Regardless of my email response in 2007, and the response from Jay Hess (and possibly others), the manuscript that I received for comment was subsequently published without the alteration of a single word.

So please excuse my lab if we insist on using the Mll and Set names rather than the defective Kmt 2 nomenclature.
